# Japan-epiretinal membrane (J-ERM) registry: A prospective cohort study protocol investigating the surgical outcome of epiretinal membrane

**DOI:** 10.1371/journal.pone.0297347

**Published:** 2024-02-08

**Authors:** Yuki Kanzaki, Ryo Matoba, Kenji Ishihara, Tetsuro Morita, Yuki Muraoka, Shuhei Kimura, Takashi Koto, Ryo Kawasaki, Takayuki Baba, Fumiki Okamoto, Makoto Inoue, Taiji Sakamoto, Akitaka Tsujikawa, Yuki Morizane

**Affiliations:** 1 Department of Ophthalmology, Okayama University Graduate School of Medicine, Dentistry and Pharmaceutical Sciences, Okayama City, Okayama, Japan; 2 Department of Ophthalmology and Visual Sciences, Kyoto University Graduate School of Medicine, Sakyo-ku, Kyoto, Japan; 3 Kyorin Eye Center, Department of Ophthalmology, Kyorin University School of Medicine, Mitaka City, Tokyo, Japan; 4 Division of Public Health, Department of Social Medicine, Osaka University Graduate School of Medicine, Suita City, Osaka, Japan; 5 Department of Ophthalmology and Visual Science, Chiba University Graduate School of Medicine, Chuo-ku, Chiba, Japan; 6 Department of Ophthalmology, Graduate School of Medicine, Nippon Medical School, Bunkyo-ku, Tokyo, Japan; 7 Department of Ophthalmology, Kagoshima University Graduate School of Medical and Dental Sciences, Kagoshima City, Kagoshima, Japan; St. Marianna University School of Medicine, JAPAN

## Abstract

**Background:**

Epiretinal membrane (ERM) causes visual impairment such as reduction in visual acuity and metamorphopsia due to retinal traction. With the improvement of optical coherence tomography (OCT) and microincision vitrectomy surgery (MIVS), the surgery of ERM has significantly advanced. However, there have been no large-scale studies on the following: (1) how to evaluate visual impairment in ERM, (2) the relationship between OCT findings and visual function, (3) when is the optimal timing of surgery, and (4) the relationship between the surgical instruments as well as techniques and prognosis. The purpose of this study was to obtain evidence regarding these ERM surgeries.

**Methods and design:**

This is a prospective, multicenter cohort study of ERM surgery in Japan from March 1, 2023, to March 31, 2027 (UMIN000048472, R-3468-2). Patients who underwent ERM surgery during the study period and agreed to participate in this study will be included. The goal is to have a total of 5,000 eyes surgically treated for ERM. The following data will be collected: age, gender, medical history, subjective symptoms, visual function before and 6 and 12 months after surgery, clinical findings, OCT data, surgical technique, instruments used in surgery, and complications.

**Discussion:**

The results of this study will support the surgical decisions and procedures in ERM practices.

## Introduction

Epiretinal membrane (ERM) is a common type of fibrocellular proliferation found on the internal limiting membrane (ILM) of the retina, and is significantly associated with aging [1–3]. The major symptoms of ERM include reduced visual acuity, metamorphopsia, and aniseikonia [4–8].

Recently, considerable improvements have been observed in optical coherence tomography (OCT) and micro incision vitrectomy surgery (MIVS) system, and ERM surgery is more advanced than ever before. However, there are still many unsolved issues involving ERM surgery, such as evaluation protocols regarding visual impairment, the relationship between OCT findings and visual function, the optimal timing of surgery, and the relationship between ERM surgical techniques as well as the instruments and prognosis, and lastly, the safety of the surgery. Although reports on each of these topics have been published from a single center or a small number of centers, no generalizable conclusions have been arrived at.

Randomized controlled trials (RCTs) are the best way to obtain high levels of evidence for treatment efficacy. However, it is difficult to conduct an RCT for a disease for which existing treatments have already been established, such as surgery. Recently, real-world data analysis with a quasi-experimental design using big data derived from day-to-day clinical practice has been conducted instead of RCTs. A report on reoperation after ERM surgery based on IRIS (Intelligent Research In Sight) registry data has been published in the United States [9]. In Japan, the Japanese Retina and Vitreous Society (JRVS) conducted a nation-wide rhegmatogenous retinal detachment (RRD) registry study and reported the prognosis of RRD surgery using big data [10–22].

In this study, with ERM as the target disease, we represent JRVS aiming to obtain a high level of evidence regarding the surgery for ERM based on a nation-wide registry, including visual function tests in ERM and the optimum timing of surgical intervention along with the OCT findings, and the relationship between surgical technique and pre- and postoperative visual functions.

## Methods

### Study design and ethical considerations

This is a prospective, multicenter cohort study of ERM surgery in Japan from March 1, 2023, to March 31, 2027. This study adheres to the Declaration of Helsinki and was approved by the Ethics Committee of Kyoto University (UMIN000048472, R-3468-2). In this study, the need to obtain written informed consent from the patients will be waived off as no randomization will be included. This study will be carried out using the opt-out approach following the Ethical Guidelines for Medical and Biological Research Involving Human Subjects.

### The sample size, inclusion, and exclusion criteria

The goal is to register a total of 5,000 eyes surgically treated for ERM. The following patients will be included in the study: (1) who undergo ERM surgery and (2) who can be followed up for 1 year after surgery, regardless of whether the ERM is idiopathic or secondary.

### Registered data

The following data will be collected: age, gender, medical history, subjective symptoms, visual function, clinical findings, OCT data, surgical technique, instruments used in surgery, and complications (Table 1). Snellen visual acuity will be measured before and 6 and 12 months after surgery and decimal best corrected visual acuity will be converted to logMAR units for analysis. The collected data will be stored securely in locked computers at each facility, anonymized, and registered via the Internet. The data center will be placed in the JRVS office, and the data will be stored and managed under utmost security. All data will be checked at least twice by two researchers. When inconsistencies or outliers are found, the committee will contact the facility to confirm them, and if they cannot be confirmed, a final decision will be made by the committee.

**Table 1:** The list of collected information of the J-ERM registry.

Preoperative characteristics	Surgical procedure	Postoperative characteristics
		(6 months/12 months postoperatively)
Age	Date of surgery	Best-corrected visual acuity
Sex	Combined cataract surgery	M-CHARTS score
Laterality	Gauge of vitrectomy instruments (23/25/27)	Contrast sensitivity
Symptoms	Adjuvants	CRT
Best-corrected visual acuity	Presence of PVD	Postoperative complications
Spherical equivalent	Forceps	
Intraocular pressure	Macular procedures performed during ERM peeling	
Axial length	Handling of EP	
M-CHARTS score	Macular staining	
Contrast sensitivity	Intraocular tamponade materials	
Lens status	Intraoperative complications	
Complication of posterior capsule opacification		
Complication of glaucoma		
Primary or secondary ERM		
Classification of ERM		
(ERM/MPH/LMH/FS)		
High myopia		
Cause of secondary ERM		
ERM of the fellow eye		
OCT device		
CRT		
ERM staging^a^		
Presence of PVD		
Complication of VMT		
Presence of EIFL		
Presence of cystoid macular edema		
Presence of ellipsoid zone disruption		
Presence of acquired vitelliform lesion		

Abbreviations: ERM, epiretinal membrane; PVD, posterior vitreous detachment; VMT, vitreomacular traction; TA, triamcinolone acetonide; EP, epiretinal proliferation; MPH, macular pseudohole; LMH, lamellar macular hole; FS, foveoschisis; OCT, optical coherence tomography; CRT, central retinal thickness.

^a^Govetto et al. AJO 2017

### Safety

Since this is an observational study, there are no risks associated with participation in this study that are more than the standard care for the treatment of ERM practice in Japan.

### Statistical analysis

An overview of the clinical picture and what treatment options are selected will be evaluated by descriptive statistics. Postoperative visual function and complications will also be examined by multivariate analysis to determine the relevant pre- and intraoperative factors. Continuous variables will be analyzed with the Mann-Whitney U test or the Kruskal-Wallis test (or with the Student’s *t-test* or one-way ANOVA for parametric tests), and categorical variables will be analyzed with the chi-squared test or Fisher’s exact test. A *P* value of < 0.05 will be considered statistically significant. Multivariate analysis will be conducted considering potential confounding findings with covariates associated with both the selection of treatment options and the outcome. In addition, statistical methods such as multilevel mixed models and quasi-comparative study designs with propensity scores and control variables will be used.

## Discussion

With improvements in the performance of diagnostic equipment and surgical instruments, ERM has become the leading macular disease for which vitrectomy is performed. However, there is no clear evidence as to which cases should be operated on or as to the efficacy and safety of surgery. Although there have been many retrospective studies on ERM, they have been conducted in a small number of facilities with a small number of cases, and the studies on ERM surgery have had short postoperative follow-up periods [23–25]. The present study is a large, multicenter, prospective, observational study in Japan, with a planned follow-up period of up to 1 year after surgery. The design of this study overcomes the shortcomings of previous studies and has the potential to establish evidence regarding ERM surgery.

Regarding the evaluation of visual impairment and the prognosis of visual function in ERM, there are many unanswered questions. Visual impairment in ERM has traditionally been assessed by visual acuity testing [23, 24]. However, even patients with good visual acuity may show metamorphopsia [5, 26] and reduced contrast sensitivity [27], and these tests are useful in the evaluation of visual function from multiple viewpoints before and after surgery in ERM. In this study, we will evaluate metamorphopsia and contrast sensitivity as well as analyze the course of each parameter both pre- and post-surgery. We also plan to collect data on the condition of the lens [27, 28] and the presence of posterior vitreous detachment (PVD) [29], which may affect various visual function tests. In addition, identifying the extent to which surgical intervention is performed for ERM at the time of visual deterioration and the factors associated with postoperative visual function may provide insight into the appropriate timing of surgical intervention. Specifically, as various OCT data, including B-scan and 3D images, will be collected from all patients in this study, we will analyze the relationships between postoperative visual functions and the staging of B-scan images [30] and the maximum depth of the retinal folds [31], thereby determining the appropriate timing of surgery.

While the morphological analysis of ERM has progressed with the development of OCT, detailed classification of ERM based on OCT findings has raised new clinical questions regarding the relationship between various OCT findings and visual function in ERM. OCT findings in ERM include ectopic inner foveal layer (EIFL) [30], macular cysts [32], EZ disruptions [33], and central foveal bouquet abnormalities [33] on B-scan images, and retinal folds on en face images [31, 34–38] constructed from 3D retinal images. Although the relationship between these findings and various visual functions and their application to the criteria for surgery have been reported [31], all of these studies were conducted in a small number of patients. On the other hand, epiretinal proliferation (EP) has recently been distinguished as a distinct lesion with OCT findings similar to those of ERM but with completely different characteristics, such as traction and histological findings [39, 40]. Based on this finding, Hubschman et al. proposed an OCT-based consensus definition of lamellar macular hole (LMH) and related diseases, classifying them into three categories: LMH, which is frequently associated with EP; ERM-Foveoschisis (FS), which traction by ERM is the primary pathology; and macular pseudohole (MPH) [41]. This study may provide insight into the postoperative prognosis of ERM, LMH, and related diseases and may lead to the discovery of OCT findings that predict postoperative visual outcomes.

The following four points are debatable and/or unknown intraoperatively and postoperatively in ERM surgery: i) whether or not ILM peeling should be performed, ii) the choice of surgical adjuvants for staining, surgical instruments (the gauge size in vitrectomy system and type of forceps), intraocular tamponade material selection; iii) the choice of how to handle EP in cases with EP; iv) the incidence of postoperative complications and factors involved in their occurrence. While ILM peeling can reduce the recurrence of ERM [42–44], ILM peeling is known to decrease retinal sensitivity [25, 45, 46]. No conclusion has been reached as to which cases should be treated with ILM peeling. Moreover, brilliant blue G, and indocyanine green, and triamcinolone acetonide are commonly used as visualization agents for membrane peeling [47–49], but they are used at the discretion of each physician, and no clear criteria exist for their use. Similarly, because no large-scale studies have been conducted, the choice of the gauge size of the vitrectomy system, the type of intraocular forceps, and the use of tamponade material (balanced salt solution, air [50, 51], and gas [52]) is at the discretion of each surgeon. In addition, various techniques have been reported for ERM cases associated with EPs, which are frequently seen in LMH, including EP removal [53, 54], EP embedding [55, 56], and ILM peeling without EP implantation [57]. However, there have been no large-scale studies on the postoperative outcomes of these techniques. Furthermore, postoperative complications of ERM include cataract progression [58], posterior capsular opacification [59, 60], epiretinal membrane recurrence [44], cystoid macular edema (CME) development [61], postoperative endophthalmitis [62, 63], retinal tears and detachment [64], and macular hole [65]. However, the incidence of each of these complications is low, and no analysis of large case series has been performed since the widespread use of MIVS, so the exact incidence and pre- and intraoperative factors associated with each complication are unknown. To address these important questions, we will employ statistical techniques like multivariate analysis and other modeling approaches, such as multilevel mixed models. In addition, statistical methods such as multilevel mixed models and quasi-comparative study designs with propensity scores and control variables will be used.

## Conclusions

This study may provide useful insights into these debates in ERM surgery.

The results of this study may lead to optimization of ERM treatment. The data from this study will assist physicians involved in ERM practice and surgery in making surgical and procedural decisions.
